# Dietary Zinc Intake and All-Cause and Cardiovascular Mortality in Korean Middle-Aged and Older Adults

**DOI:** 10.3390/nu15020358

**Published:** 2023-01-11

**Authors:** Yu-Jin Kwon, Hye Sun Lee, Goeun Park, Juyeon Yang, Hyung-Mi Kim, Ji-Won Lee

**Affiliations:** 1 Department of Family Medicine, Yongin Severance Hospital, Yonsei University College of Medicine, Yongin 16995, Republic of Korea; 2 Biostatistics Collaboration Unit, Department of Research Affairs, Yonsei University College of Medicine, Seoul 06273, Republic of Korea; 3 Biomedical Statistics Unit, Research Institute for Future Medicine, Samsung Medical Center, Seoul 06351, Republic of Korea; 4 Department of Food and Nutrition, Dongduk Women’s University, Seoul 02748, Republic of Korea; 5 Department of Family Medicine, Severance Hospital, Yonsei University College of Medicine, Seoul 03722, Republic of Korea

**Keywords:** dietary zinc intake, all-cause mortality, cancer mortality, cardiovascular disease mortality, cohort study

## Abstract

We aimed to investigate the association between dietary zinc intake and total deaths, cancer, and cardiovascular disease death. In this prospective, 10-year, community-based cohort analysis, data from 143,050 adult participants (aged 40 years and older) were analyzed. Dietary zinc intake at baseline was assessed using a food frequency questionnaire. Harrell’s C-index was used to determine the optimal cut-off of dietary zinc intake with the log-rank test. Using the Cox proportional hazards regression models, the association between dietary zinc intake and all-cause, cancer, and cardiovascular disease mortality was estimated using hazard ratios and 95% confidence intervals. During the mean 10.1 years of follow-up, 5436 participants expired, of whom 2355 died due to cancer and 985 died due to cardiovascular causes. After adjustment for confounders, dietary zinc intake was inversely associated with all-cause mortality (≤5.60 mg/day vs. >7.98 mg/day; hazard ratio, 1.13; 95% confidence interval, 1.01–1.25) and cardiovascular disease mortality (≤5.12 mg/day vs. >7.28 mg/day; hazard ratio, 1.42; 95% confidence interval, 1.11–1.81) but not with cancer mortality (≤5.60 mg/day vs. >10.08 mg/day; hazard ratio, 1.09; 95% confidence interval, 0.90–1.33). Dietary zinc intake was associated with a lower risk of all-cause mortality and cardiovascular disease mortality but not with cancer mortality. Our findings could suggest that recommending optimal dietary zinc intake is helpful for human health.

## 1. Introduction

Micronutrients are needed to deliver energy and maintain homeostasis in the human body [1]. Zinc is an essential trace mineral important for the formation and function of structural proteins, enzymatic processes, and transcription factors [2]. It is also involved in various metabolisms, including antioxidant and anti-inflammatory activities and immune modulation [3], which when dysregulated may lead to age-related degenerative diseases such as infections, atherosclerosis, and cancer [4].

Regular zinc intake through diet is required to maintain a steady zinc status because the human body cannot produce and store zinc [5]. Animal products, including red meat, poultry, and oysters, are rich in zinc, whereas plant-based sources of zinc are whole grains, beans, and nuts [6]. The bioavailability of zinc from plant-based foods is lower than that of animal-based foods due to the presence of several inhibitors such as phytic acid, calcium, and polyphenols [2]. However, the bioavailability of zinc could be increased by food-processing methods such as soaking, heating, sprouting, fermenting and leavening [7].

Although the optimal level of zinc remains controversial, the daily requirement for zinc for adults ranges from 7–11 mg [8]. However, zinc deficiency is highly prevalent worldwide (~7.5–30%) [9], and in some regions, rates of deficiency reach 60–70% [10]. Due to the plausible biological functions of zinc in the human body, insufficient dietary zinc intake and the consequences of zinc deficiency could increase mortality and morbidity [2].

Previous studies have reported that inconsistent relationship between dietary zinc intake and mortality [11,12,13,14]. The conflicting results might be due to the differences in food sources, food processing, and average zinc intake according to sex, race, and other population [15]. Most studies were conducted in Western countries, and only a few studies have explored the longitudinal association between dietary zinc and mortality in Asian populations. Additionally, no study considered the cut-off value ranges of dietary zinc to maximize the predictive ability for mortality.

Therefore, in this study, we divided dietary zinc intake according to optimal cut-off value ranges derived using Harrell’s C-index and investigated the association between dietary zinc intake and all-cause, cancer, and CVD mortality using a large cohort database representative of the Korean population.

## 2. Materials and Methods

### 2.1. Participants

We analyzed the baseline data of adult participants, who were aged ≥40 years from the Korean Genome and Epidemiology Study (KoGES), Ansan-Ansung Study (2001–2002), KoGES Health Examinee Study (2004–2013), KoGES Cardiovascular Disease Association Study (2005–2011). KoGES is a large-scale, longitudinal, and prospective cohort studies. Detailed information regarding KoGES is described in a previous study [16] and is available on the following website: https://www.kdca.go.kr/contents.es?mid=a40504010000 (accessed on 20 May 2022).

**Figure 1:**
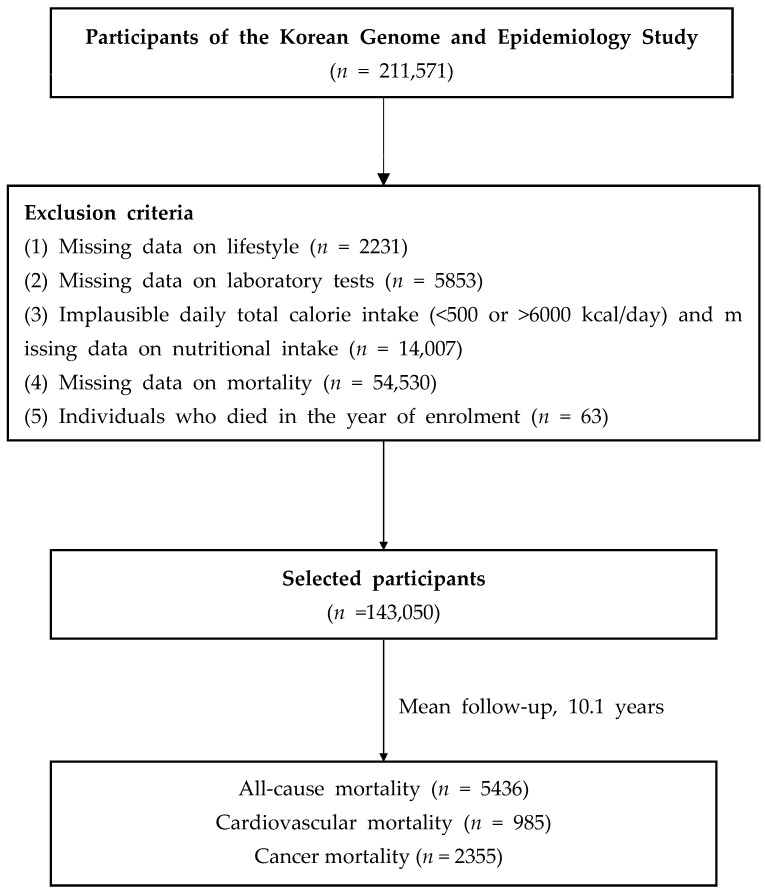
Flow chart of the study population.

Of the 211,571 participants in the baseline survey (2001–2013), we included 143,050 participants after excluding 68,521 participants who lacked the following data: (1) age and lifestyle factors (*n* = 2231); (2) laboratory test results (*n* = 5853); (3) dietary information and implausible total calorie intake (<500 or >6000 kcal/day; *n* = 14,007); (4) mortality (*n* = 54,530); (5) individuals who died in the year of enrolment (*n* = 63) (Figure 1). All participants provided informed consent. This study was approved by the institutional review board (IRB) of Yongin Severance Hospital (IRB number: 3-2020-0043).

### 2.2. Dietary Assessment

Dietary intake was assessed using a semiquantitative food frequency questionnaire (FFQ), administered by a trained interviewer, which was developed for a community-based cohort of the KoGES. FFQ assessed the frequency of consumption of each food per participant in the past year.

Although FFQ contains a limited number of food items and lacks in accuracy in individuals’ food record, it is a practical dietary assessment tool used in most prospective cohort studies. The FFQ involved 103 food items and was validated in the previous studies [17]. In the current study, FFQ includes the following foods; rice and other cereals, noodles and bread, vegetables, potatoes, mushrooms, soybean, soybean products, and other beans, common fish, other fish and shellfish, meats, seaweeds, eggs, milk and dairy products, fruits, beverages, snacks, nuts, and fats. The serving portion was determined by the median value of each food item using the data from the Korea National Health and Nutrition Examination Survey which is composed of a representative sample of the Korean population. The serving portions were classified as follows: small, medium and large. To help participants’ understanding for serving size, pictures on serving size for food items were provided. The frequency of food intake was categorized into nine as follows; never or seldom, once a month, 2–3 times a month, one to two times a week, three to four times a week, five to six times a week, once a day, twice a day or three times or more every day. Nutrient intake was converted based on the weight derived from the food intake frequency and portion size. More detailed information is available in the KoGES website https://www.kdca.go.kr/contents.es?mid=a40504010000 (accessed on 20 May 2022).

### 2.3. Covariates

All health examination procedures were performed by trained medical staff. Blood pressures were measured twice with participants in the seated position. Blood tests were conducted after 8 h fasting. Serum glucose, hemoglobin A1C, total cholesterol, high-density lipoprotein, and triglyceride levels were enzymatically determined using a Chemistry Analyzer (Hitachi 7600, Tokyo, Japan until August 2002, and ADVIA 1650, Siemens, Tarrytown, NY from September 2002). Smoking (current, former, and never smoker), alcohol intake (current, former, and never drinker), and physical activity (regular exerciser was defined as individuals who regularly exercised until sweating) were self-reported through the questionnaire. Hypertension was defined a systolic blood pressure (SBP) of 140 mm Hg or more, or a diastolic blood pressure (DBP) of 90 mm Hg or more, or taking medication. Diabetes was defined as a fasting glucose of 126 mg/dL or more, or hemoglobin A1C of 6.5% or more, or taking medication. Dyslipidemia was defined as serum total cholesterol of 200 mg/dL or more, or serum triglyceride of 150 mg/dL or more, or taking medication. Chronic kidney disease (CKD) was defined as glomerular filtration rate (GFR) < 60 mL/min/1.73 m^2^ (calculated using the Chronic Kidney Disease Epidemiology Collaboration equation).

### 2.4. Study Outcomes

Mortality status was ascertained through data linkage based on the unique personal identification key code system as KoGES data are linked to the national data sources (Korea National Statistical Office), including mortality records. Participants were followed from the data of the baseline survey to the time of mortality event, or end date of the study, or the date of last contact. Participant mortality was tracked from January 2001 to December 2019, and mortality causes were classified based on the International Classification of Diseases (ICD) codes listed in the National Mortality Index. All-cause mortality included all deaths of specified and unknown causes, cancer mortality includes deaths under ICD-10 codes C00–C97, and CVD mortality includes deaths under ICD-10 codes I00–I99.

### 2.5. Statistical Analysis

Data are presented as means (standard deviation [SD]) and number (percentage). Participants were divided into three groups based on optimal cut-off points. The optimal cut-off range of dietary zinc intake (mg/day) for clinical outcomes was determined using Harrell’s C-index (Supplementary Figure S1–S3). The optimal cut-off point was selected by maximizing the ability to predict mortality. It was applied similarly to sex and other outcomes, including cancer and CVD mortality. The baseline characteristics of participants according to mortality status were compared using the one-way analysis of variance for continuous variables or the chi-square test for categorical variables. The survival rates according to dietary zinc intake were estimated using Kaplan–Meier curves and log-rank tests. The warranty period, defined as the required time for the cumulative incidence to reach 0.5%, and incidence per 1000 person-years were calculated. Univariable and multivariable Cox proportional hazards regression models were constructed to assess the independent relationship of dietary zinc intake to outcomes. In multivariable Cox models, we adjusted variables with *p* < 0.1 on the univariate analysis and previously reported variables, including age, sex, body mass index (BMI), alcohol intake, smoking, regular exercise, total calorie intake, dyslipidemia, hypertension, diabetes, and CKD. All statistical analyses were performed using SAS 9.2 (SAS Institute, Cary, NC, USA). Two-sided *p*-values < 0.05 were considered statistically significant.

## 3. Results

Of the 14,030 participants, there were 5436 all-cause deaths, 2355 cancer deaths, and 985 CVD deaths during the 10-year follow-up period (Table 1). Baseline characteristics of the study population according to the mortality status (all-cause, cancer, and CVD mortality) are presented in Table 1. We initially found a negative association between dietary zinc intake (continuous value) and all-cause, cancer, and CVD mortality (data not shown). The dietary zinc intake (mg/day) optimal cutoff ranges, which maximized the Harrell’s C-index values of all-cause mortality in all participants, men, and women were: (1) all; ≤5.60, 5.60–7.98, and >7.98, (2) Men; ≤5.60, 5.60–7.23, and >7.23, (3) Women; ≤5.48, 5.48–7.99, and >7.99 (Figure S1).

Dietary zinc intake (mg/day) optimal cut-off points, which maximized Harrell’s C-index values of cancer and CVD mortality in all, men, and women are presented in Figure S2 (cancer mortality) and Figure S3 (CVD mortality). Baseline characteristics of the entire study population according to dietary zinc cut-off values are presented in Table S1. Participants who consumed zinc of >7.98 mg/day were likely men, younger, with higher BMI and WC, had a higher prevalence of current smoker and alcohol drinking status, and participated in regular exercise; however, they showed a lower prevalence of hypertension and CKD. Participants who consumed zinc of >7.98 mg/day consumed higher total energy intake, carbohydrates (g/day), proteins (g/day), and fats (g/day). Baseline characteristics of the study population according to dietary zinc intake cut-off values by sex are presented in Tables S2 (men) and S3 (women).

**Figure 2:**
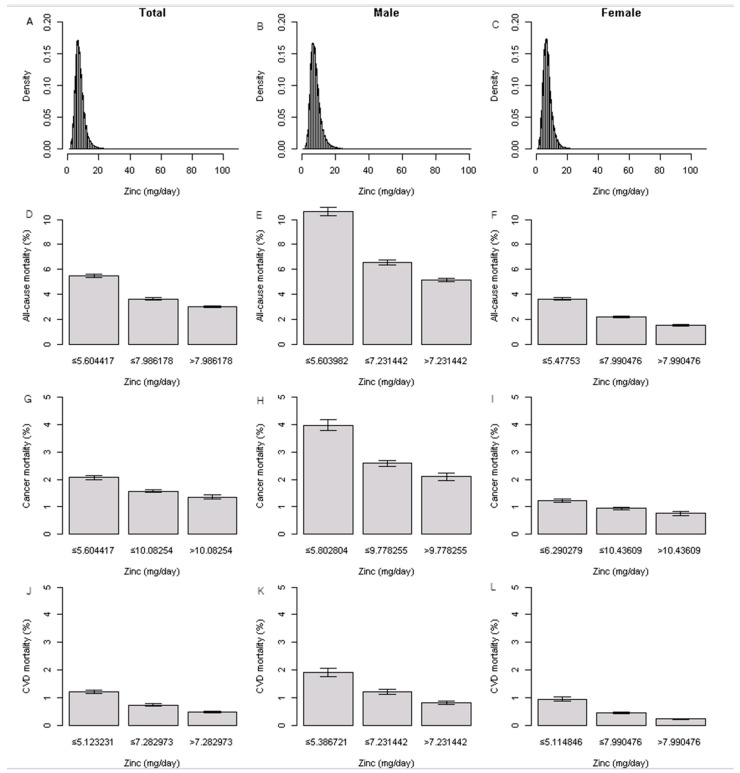
Distribution of dietary zinc intake and proportions of all-cause, cancer, and CVD mortality.

Figure 2A–L presents the dietary zinc intake density plots in all, men, and women. The mean dietary zinc intake was 7.86 ± 3.55 mg/day in all participants, 8.29 ± 3.56 mg/day in men, and 7.62 ± 3.52 mg/day in women. The proportions of all-cause, cancer, and CVD mortality are shown in Figure 2. The prevalence of all-cause, cancer, and CVD mortality was significantly higher in those who consumed the lowest zinc intake among all participants, men, and women (all, *p* < 0.001; Table S4).

Table 2 presents the warranty period of all-cause, cancer, and CVD mortality according to dietary zinc intake cutoff values in all participants, men, and women. All-cause, cancer, and CVD mortality were associated with the longest 0.5% warranty period of 3.4 years for participants with zinc >7.98 mg/day intake group, 5.3 years for those with zinc >10.08 mg/day intake group, and 10.6 years for those with zinc >7.28 mg/day intake group. In men, all-cause, cancer, and CVD mortality were associated with the longest 0.5% warranty period of 2.3 years for participants with zinc >7.23 mg/day intake group, 3.9 years for those with zinc >9.78 mg/day intake group, and 7.7 years for those with zinc >7.23 mg/day intake group. In women, all-cause, cancer, and CVD mortality were associated with the longest 0.5% warranty period of 5.2 years for participants with zinc >7.99 mg/day intake group, 7.9 years for those with zinc >10.44 mg/day intake group, and 13.5 years for those with zinc >7.99 mg/day intake group.

The incidence rates for all-cause, cancer, and CVD mortality were the lowest for participants with a zinc intake of >7.98 mg/day (2.91 per 1000 person-years; 95% confidence intervals [CI], 2.46–3.36), followed by those with a zinc intake of >10.08 mg/day (1.31 per 1000 person-years; 95% CI, 0.86–1.76), and those with a zinc intake of >7.28 mg/day (0.47 per 1000 person-years; 95% CI, 00.31–0.63). Similar trends were observed in men and women.

Table 3 summarizes the association between baseline dietary zinc intake and all-cause, cancer, and CVD mortality of the study cohort. In the unadjusted model, low dietary zinc intake was associated with higher all-cause, cancer, and CVD mortality (all-cause mortality: ≤5.60 mg/day vs. >7.98 mg/day, hazard ratio [HR], 1.80; 95% CI, 1.68–1.93; cancer mortality: ≤5.60 mg/day vs. >10.08 mg/day; HR, 1.50; 95% CI, 1.32–1.71; CVD mortality: ≤5.12 mg/day vs. >7.28 mg/day; HR, 2.42; 95% CI, 2.07–2.84). Similar trends were observed in men and women. In the fully adjusted model (adjusted for age, sex, BMI, smoking, alcohol consumption, exercise, total calorie intake, hypertension, diabetes, dyslipidemia, and CKD), lower zinc intake was significantly associated with all-cause and CVD mortality (≤5.60 mg/day vs. >7.98 mg/day; HR, 1.13; 95% CI, 1.01–1.25 for all-cause mortality; and ≤5.12 mg/day vs. >7.28 mg/day; HR, 1.42; 95% CI, 1.11–1.81 for CVD mortality) but not cancer mortality (≤5.60 mg/day vs. >10.08 mg/day; HR, 1.09; 95% CI, 0.90–1.33 ). Similar trends were observed in men and women.

## 4. Discussion

In this large, population-based, prospective cohort study of middle-aged and older adults, we found that dietary zinc intake of >7.98 mg/day and >7.28 mg/day was associated with a lower risk of all-cause and CVD mortality in all participants, respectively. In the sex-specific analysis, dietary zinc intake of >7.23 mg/day and >7.99 mg/day were significantly associated with a lower risk of all-cause and CVD mortality in men and women, respectively. However, dietary zinc intake was not significantly associated with cancer mortality.

Our findings are consistent with those from previous studies that showed an inverse association between dietary zinc intake and all-cause and CVD mortality [11,12,18]. The British National Diet and Nutrition Survey with participants aged over 65 years reported that total mortality was reduced by the increment of plasma zinc concentrations (per SD) (HR, 0.79; 95 % CI 0, 0.72–0.87) and dietary zinc of food energy (per SD) (HR, 0.86; 95 % CI, 0.79–0.94) [11]. In the Iowa Women’s Health Study, the relative risk with 95% CI for CVD mortality in the highest versus the lowest quintiles of zinc intake was 0.37 (0.13, 1.06) (*p* for trend = 0.07) [12]. A Japanese study showed that the adjusted HRs with 95% CIs for coronary heart disease mortality in the highest versus the lowest quintiles of zinc intake were 0.68 (0.58–1.03; *p*-trend = 0.05) [18]. Furthermore, several studies have shown that zinc deficiency is associated with an increased risk of CKD [19], diabetes [20], and CVD [21,22]. 

Zinc has an important role in maintaining human health through its anti-oxidant and anti-inflammatory effects [23]. Zinc deficiency contributed to endothelial damage and increased CVD risk through disruption of nitric oxide production and increase in oxidative stress [23]. Zinc is also an important role in immune system modulation. Zinc deficiency leads to a dysregulation of interleukin-10 (IL)-10, IL-2, and IFN-γ production [24]. A recent systemic review and meta-analysis reported that zinc supplements lowered mortality in 2019 coronavirus disease COVID-19 patients [25]. Inadequate zinc intake is the primary determinantal reason for zinc deficiency worldwide [26], and it is not surprising that zinc deficiency is associated with a higher risk of all-cause and CVD mortality. 

However, there are also contrary findings regarding zinc status and chronic disease and mortality in the literature [13,14]. The Australian Longitudinal Study on Women’s Health (ALSWH) found that the highest zinc-to-energy ratio quintiles was associated with an increased incidence of CVD in middle-aged women (odds ratio, 1.67 and 95% CI, 1.08–2.62) than those with the lowest quintile [27]. A Chinese study reported that the highest quartile of dietary zinc to energy ratio was associated with increased all-cause and cancer mortality [14]. Although the exact reasons for these discrepancies are unclear, several approaches were used to examine the relationship between dietary zinc and mortality.

The recommended dietary intake (RDI) of zinc for Korean adults is 11 mg/day for males and 8 mg/day for females, which are similar to the Chinese, Japanese, and USA RDIs of zinc [6,27,28,29]. In the current study, zinc intake was insufficient in 84.8% of men and 64.8% of women aged over 40 years. Similarly, in Japan, zinc intake is insufficient in 60%–70% of both genders aged over 20 years [10]. However, zinc excess is harmful as it may result in cellular oxidative stress [30,31]. Interestingly, a higher serum zinc level was associated with a higher risk of diabetes in a systemic review [20]. Furthermore, relatively higher zinc intake tends to have a negative impact on mortality. In the ALSWH study, the dietary zinc intake of the highest quintiles was 17.35 mg/day (95% CI, 17.12–17.59) with a mean intake of 10.66 mg/day [27]. In a Chinese study, the mean zinc intake was 13.3 (SD, 3.7) mg/day in men, 11.0 (SD, 3.2) mg/day in women, and in total, 47.1% of men and 81.7% of women had an estimated zinc intake above RDI [20]. Therefore, optimal zinc intake is essential to maintaining individuals’ health.

Most previous studies classified dietary zinc intake into quintiles or continuous values. However, clinical outcomes, including mortality, may be affected by zinc intake in a non-linear or non-monotonic relationship [32]. Therefore, we tried to determine the optimal cut-off value ranges of dietary zinc intake rather than the linear response of zinc intake to boost the potential discriminatory ability for mortality. Indeed, when we divided dietary zinc intake into quintiles, we found an inverse association between dietary zinc intake and all-cause and CVD mortality in an unadjusted model (Q1 vs. Q5 (reference); HR, 1.90; 95% CI, 1.75–2.06; *p* < 0.001 and Q1 vs. Q5; HR, 2.35; 95% CI, 1.93–2.88; *p* < 0.001). However, this association was weakened after adjusting for potential confounders. We also identified an inverse association between dietary zinc to energy ratio and all-cause and CVD mortality (data not shown).

Another possible reason for these conflicting results between zinc intake and morality might be due to the food sources of dietary zinc. It is possible that different sources of dietary zinc have different effects on the risks of mortality. In the Multi-Ethnic Study of Atherosclerosis, intake of zinc, from red meat but not from other sources, was associated with a greater risk of CVD and metabolic syndrome [15]. A typical Korean diet consists of phytate-rich foods with low zinc content, such as grains, cereals, legumes, and vegetables, which may lower zinc absorption [33]. Therefore, the bioavailability of zinc intake could be lower than in other ethnic groups. Additionally, other components in plant food could lead to beneficial effects on health.

In our study, dietary zinc intake was not associated with cancer mortality. Although the exact cause is still unclear, zinc concentration varies in organs, from up to 200 µg/g in the pancreas, prostate, and bone to 1 µg/g in the plasma and brain, and the effects of zinc on carcinogenesis appear to be site-specific [34]. Further studies are needed to clarify the association between zinc intake and cancer-specific mortality.

Our study has several limitations. First, data regarding dietary zinc intake was derived from the FFQ, which has a disadvantage due to the lack of accuracy of absolute nutrient values, especially micronutrients, and a possibility of over- and under-reporting of certain foods [35]. However, FFQ is a useful and feasible method for application in large epidemiological cohort studies [35]. Second, the dietary assessment was conducted only at the baseline survey. Therefore, the time-varying effects of dietary zinc intake and any changes in dietary habits during the follow-up period could not be reflected in the current study. Third, this integrated three KoGES datasets provided only 23 nutrients derived from the FFQ. Therefore, we could not distinguish the sources of dietary zinc. Forth, serum zinc concentrations were not available in the KoGES, although serum zinc concentration cannot fully reflect the participant’s zinc status [36]. Fifth, we could not obtain information on the use of zinc supplements. Further studies should consider the effect of zinc supplements. Finally, we included middle-aged and older Korean adults, which limits the generalizability of our results to other countries and ethnic groups.

Despite these limitations, our study has several strengths. This is a large population-based cohort study, and the numbers of mortality were relatively larger than in other studies. Furthermore, we divided dietary zinc intake according to the optimal cut-off value ranges to increase the discriminatory ability for predicting mortality. We also conducted a sex-specific analysis and found that sex-specific cut-off points of dietary zinc intake were associated with all-cause and CVD mortality.

## 5. Conclusions

We found that dietary zinc intake was associated with a lower risk of all-cause and CVD mortality but not with cancer mortality. Further investigations into the biological mechanisms of zinc’s action on the pathogenesis of mortality are required. Moreover, additional studies are needed detailing the source of dietary zinc and its precise roles.

## Figures and Tables

**Table 1:** Baseline characteristics of the study population according to mortality status.

Variables	Alive	Total Death	CVD Death	Cancer Death	P1	P2	P3
N	137,614	5436	985	2355			
Sex (men)	47,640 (34.6)	3304 (60.8)	558 (56.7)	1405 (59.7)	<0.001	<0.001	<0.001
Age (years)	53.5 ± 8.5	62.8 ± 9.3	64.9 ± 9.2	61.4 ± 8.7	<0.001	<0.001	<0.001
Body mass index (kg/m^2^)	24.0 ± 2.9	23.9 ± 3.2	24.1 ± 3.3	24.0 ± 3.1	0.03	0.31	0.97
WC (cm)	81.2 ± 8.6	84.1 ± 9.0	84.8 ± 9.2	83.9 ± 8.6	<0.001	<0.001	<0.001
SBP (mmHg)	122.5 ± 15.3	127.2 ± 17.2	130.6 ± 18.1	125.8 ± 16.2	<0.001	<0.001	<0.001
DBP (mmHg)	76.2 ± 10.0	77.5 ± 10.4	78.8 ± 10.9	77.5 ± 10.2	<0.001	<0.001	<0.001
Glucose (mg/dL)	95.3 ± 20.6	104.3 ± 34.5	105.0 ± 34.8	102.2 ± 31.1	<0.001	<0.001	<0.001
HbA1c (%)	5.7 ± 0.7	6.0 ± 1.2	6.1 ± 1.1	6.0 ± 1.0	<0.001	<0.001	<0.001
TC (mg/dL)	197.6 ± 35.5	191.3 ± 39.0	194.8 ± 39.0	190.3 ± 37.4	<0.001	0.03	<0.001
HDL-C (mg/dL)	52.8 ± 13.0	48.4 ± 13.2	47.0 ± 11.7	49.0 ± 13.2	<0.001	<0.001	<0.001
Triglycerides(mg/dL)	128.9 ± 90.1	144.1 ± 107.5	148.9 ± 94.9	136.3 ± 93.7	<0.001	<0.001	<0.001
Smoking status, *n* (%)					<0.001	<0.001	<0.001
Never smoker	100,083 (72.7)	2704 (49.7)	515 (52.3)	1165 (49.5)			
Former smoker	20,476 (14.9)	1409 (25.9)	225 (22.8)	608 (25.8)			
Current smoker	17,055 (12.4)	1323 (24.3)	245 (24.9)	582 (24.7)			
Alcohol intake, *n* (%)					<0.001	<0.001	<0.001
Never drinker	70,007 (50.9)	2365 (43.51)	458 (46.5)	1002 (42.6)			
Former drinker	5221 (3.8)	564 (10.4)	88 (8.9)	243 (10.3)			
Current drinker	62,386 (45.3)	2507 (46.1)	439 (44.6)	1110 (47.1)			
Regular exercise (Yes)	69,640 (50.6)	2089 (28.4)	327 (33.2)	1000 (42.5)	<0.001	<0.001	<0.001
Hypertension, *n* (%)	22,996 (16.7)	1411 (26.0)	324 (32.9)	530 (22.5)	<0.001	<0.001	<0.001
Diabetes, *n* (%)	9526 (6.9)	838 (15.4)	153 (15.5)	321 (13.6)	<0.001	<0.001	<0.001
Dyslipidemia, *n* (%)	78,211 (56.8)	3008 (55.3)	591 (60.0)	1252 (53.2)	0.03	0.05	<0.001
CKD, *n* (%)	3290 (2.4)	602 (11.1)	149 (15.1)	173 (7.4)	<0.001	<0.001	<0.001
Residential area, *n* (%)					<0.001	<0.001	<0.001
Urban	120,808 (87.8)	3548 (65.3)	565 (57.4)	1721 (73.1)			
Rural	16,806 (12.2)	1888 (34.7)	420 (42.6)	634 (26.9)			
Total energy (kcal/day)	1739.6 ± 542.0	1637.2 ± 531.7	1607.5 ± 558.6	1677.9 ± 516.8	<0.001	<0.001	<0.001
Carbohydrate (g/day)	310.1 ± 89.8	297.7 ± 87.0	293.6 ± 88.4	303.5 ± 85.8	<0.001	<0.001	<0.001
Fat (g/day)	27.5 ± 17.2	23.6 ± 16.9	22.7 ± 19.4	24.8 ± 16.1	<0.001	<0.001	<0.001
Protein (g/day)	58.7 ± 24.8	53.3 ± 24.9	51.7 ± 28.4	55.3 ± 24.5	<0.001	<0.001	<0.001
Calcium (mg/day)	439.9 ± 259.7	384.6 ± 245.1	364.5 ± 243.3	405.4 ± 247.8	<0.001	<0.001	<0.001
Phosphorus (mg/day)	884.7 ± 350.2	810.3 ± 346.7	785.7 ± 370.8	841.3 ± 336.9	<0.001	<0.001	<0.001
Iron (mg/day)	9.9 ± 4.9	8.8 ± 4.7	8.2 ± 4.5	9.3 ± 4.7	<0.001	<0.001	<0.001

WC, Waist circumference; SBP, Systolic blood pressure; DBP, Diastolic blood pressure; HbA1c, TC, Total cholesterol; HDL-C, High-density lipoprotein cholesterol; CKD, Chronic kidney disease; CVD, cardiovascular disease. Data are presented as a mean ± standard deviation or as a number (percentage). *p*-values are derived from the independent *t*-test for continuous variables and the chi-square test for categorical variables; significance was set at *p* < 0.05. P1: Alive versus total death, P2: Alive versus CVD death, P3: Alive versus Cancer death.

**Table 2:** Warranty periods for mortality according to zinc intake (mg/day).

All	Warranty Period (0.5%)	*n*	Person Time (Years)	Events, *n* (%)	Incidence per 1000 Person-Years (95% CI)
All-cause mortality					
≤5.60	2.419	32,992	338,516.06	1801(0.05)	5.32 (4.54–6.11)
5.60–7.98	3.169	55,029	560,702.29	1997 (0.04)	3.56 (3.06–4.06)
>7.98	3.414	55,029	562,418.91	1638 (0.03)	2.91 (2.46–3.36)
Cancer mortality					
≤5.60	4.501	32,992	343,158.26	683 (0.02)	1.99 (1.51–2.47)
5.60–10.08	5.086	85,044	872,887.87	1333 (0.02)	1.53 (1.27–1.79)
>10.08	5.253	25,014	258,246	339 (0.01)	1.31 (0.86–1.76)
CVD mortality					
≤5.12	6.334	22,985	240,241.34	278 (0.01)	1.16 (0.72–1.60)
5.12–7.28	9.17	50,025	517,831.53	370 (0.01)	0.72 (0.48–0.95)
>7.28	10.581	70,040	722,143.01	337 (0)	0.47 (0.31–0.63)
Men					
All-cause mortality					
≤5.60	1.663	8943	90,617.46	953 (0.11)	10.52 (8.40–12.63)
5.60–7.23	2.244	13,500	137,222.9	882 (0.07)	6.43 (5.08–7.78)
>7.23	2.337	28,501	289,015.36	1469 (0.05)	5.08 (4.26–5.91)
Cancer mortality					
≤5.80	2.412	10,444	108,564.47	416 (0.04)	3.832 (2.647–5.017)
5.80–9.78	3.83	28,500	292,679.04	737 (0.03)	2.518 (1.936–3.100)
>9.78	3.915	12,000	123,558.58	252 (0.02)	2.040 (1.232–2.847)
CVD mortality					
≤5.39	5.251	7443	78,401.15	142 (0.02)	1.81 (0.85–2.78)
5.39–7.23	7	15,000	156,001.42	182 (0.01)	1.17 (0.62–1.71)
>7.23	7.666	28,501	294,245.34	234 (0.01)	0.80 (0.47–1.12)
Women					
All-cause mortality					
≤5.48	3.418	22,098	227,610.93	804 (0.04)	3.53 (2.75–4.32)
5.48–7.99	4.334	37,503	383,470.95	824 (0.02)	2.15 (1.68–2.62)
>7.99	5.249	32,505	333,699.67	504 (0.02)	1.51 (1.09–1.93)
Cancer mortality					
≤6.29	6	34,598	358,738.58	428 (0.01)	1.19 (0.83–1.56)
6.29–10.44	7.169	45,007	461,418.94	427 (0.01)	0.93 (0.64–1.21)
>10.44	7.915	12,501	129,332.53	95 (0.01)	0.74 (0.26–1.21)
CVD mortality					
≤5.11	7.5	17,098	178,237.08	162 (0.01)	0.91 (0.46–1.36)
5.11–7.99	11.165	42,503	437,925.76	192 (0)	0.44 (0.24–0.64)
>7.99	13.496	32,505	335,405.14	73 (0)	0.22 (0.06–0.38)

CI, confidence interval; CVD, cardiovascular disease.

**Table 3:** Hazard ratios and 95% confidence intervals for all-cause mortality, cancer mortality, and cardiovascular mortality according to dietary zinc intake (mg/day).

	All		Men		Women
All-Cause Mortality	HR (95% CI)		HR (95% CI)		HR (95% CI)
Unadjusted					
≤5.60	1.80 (1.68–1.93)	≤5.60	2.04 (1.88–2.21)	≤5.48	2.31 (2.06–2.58)
5.60–7.98	1.22 (1.14–1.30)	5.60–7.23	1.25 (1.15–1.36)	5.48–7.99	1.42 (1.27–1.59)
>7.98	Ref	>7.23	Ref	>7.99	Ref
Model 1					
≤5.60	1.34 (1.25–1.43)	≤5.60	1.36 (1.25–1.48)	≤5.48	1.33 (1.18–1.49)
5.60–7.98	1.08 (1.01–1.15)	5.60–7.23	1.03 (0.95–1.12)	5.48–7.99	1.15 (1.03–1.28)
>7.98	Ref	>7.23	Ref	>7.99	Ref
Model 2					
≤5.60	1.12 (1.01–1.24)	≤5.60	1.12 (1.00–1.27)	≤5.48	1.18 (0.99–1.41)
5.60–7.98	1.00 (0.92–1.08)	5.60–7.23	0.95 (0.86–1.04)	5.48–7.99	1.10 (0.96–1.25)
>7.98	Ref	>7.23	Ref	>7.99	Ref
Model 3					
≤5.60	1.13 (1.01–1.25)	≤5.60	1.15 (1.02–1.29)	≤5.48	1.16 (0.98–1.38)
5.60–7.98	1.00 (0.93–1.08)	5.60–7.23	0.95 (0.86–1.05)	5.48–7.99	1.09 (0.96–1.25)
>7.98	Ref	>7.23	Ref	>7.99	Ref
Cancer mortality					
Unadjusted					
≤5.60	1.50 (1.32–1.71)	≤5.80	1.86 (1.59–2.18)	≤6.29	1.62 (1.29–2.02)
5.60–10.08	1.17 (1.04–1.31)	5.80–9.78	1.23 (1.07–1.42)	6.29–10.44	1.27 (1.01–1.58)
>10.08	Ref	>9.78	Ref	>10.44	Ref
Model 1					
≤5.60	1.17 (1.02–1.34)	≤5.80	1.22 (1.04–1.43)	≤6.29	1.15 (0.91–1.44)
5.60–10.08	1.07 (0.95–1.21)	5.80–9.78	1.07 (0.93–1.24)	6.29–10.44	1.14 (0.91–1.42)
>10.08	Ref	>9.78	Ref	>10.44	Ref
Model 2					
≤5.60	1.08 (0.88–1.31)	≤5.80	1.06 (0.84–1.35)	≤6.29	1.20 (0.87–1.66)
5.60–10.08	1.05 (0.91–1.21)	5.80–9.78	1.03 (0.86–1.22)	6.29–10.44	1.19 (0.92–1.53)
>10.08	Ref	>9.78	Ref	>10.44	Ref
Model 3					
≤5.60	1.09 (0.90–1.33)	≤5.80	1.08 (0.85–1.38)	≤6.29	1.20 (0.87–1.65)
5.60–10.08	1.06 (0.92–1.22)	5.80–9.78	1.03 (0.87–1.23)	6.29–10.44	1.19 (0.92–1.54)
>10.08	Ref	>9.78	Ref	>10.44	Ref
CVD mortality					
Unadjusted					
≤5.12	2.42 (2.07–2.84)	≤5.39	2.21 (1.79–2.72)	≤5.11	4.10 (3.11–5.40)
5.12–7.28	1.52 (1.31–1.76)	5.39–7.23	1.45 (1.19–1.76)	5.11–7.99	2.01 (1.53–2.63)
>7.28	Ref	>7.23	Ref	>7.99	Ref
Model 1					
≤5.12	1.50 (1.27–1.78)	≤5.39	1.40 (1.13–1.74)	≤5.11	1.69 (1.26–2.25)
5.12–7.28	1.22 (1.05–1.42)	5.39–7.23	1.19 (0.98–1.44)	5.11–7.99	1.40 (1.07–1.84)
>7.28	Ref	>7.23	Ref	>7.99	Ref
Model 2					
≤5.12	1.43 (1.12–1.83)	≤5.39	1.37 (1.01–1.85)	≤5.11	1.70 (1.11–2.62)
5.12–7.28	1.22 (1.02–1.46)	5.39–7.23	1.21 (0.96–1.52)	5.11–7.99	1.44 (1.04–1.99)
>7.28	Ref	>7.23	Ref	>7.99	Ref
Model 3					
≤5.12	1.42 (1.11–1.81)	≤5.39	1.38 (1.02–1.87)	≤5.11	1.66 (1.08–2.56)
5.12–7.28	1.21 (1.01–1.45)	5.39–7.23	1.21 (0.96–1.53)	5.11–7.99	1.43 (1.03–1.98)
>7.28	Ref	>7.23	Ref	>7.99	Ref

Model 1: Adjusted age, sex, and body mass index. Model 2: Adjusted age, sex, body mass index, smoking, alcohol consumption, exercise, and total calorie intake. Model 3: Adjusted age, sex, body mass index, smoking, alcohol consumption, exercise, total calorie intake, hypertension, diabetes, dyslipidemia, and chronic kidney disease. CI, confidence interval; CVD, cardiovascular disease; HR, hazard ratios.

## Data Availability

Data described in the manuscript, and code book will be made publicly and freely available without restriction at the following website: https://www.kdca.go.kr/contents.es?mid=a40504010000 (accessed on 20 April 2022).

## Supplementary Materials

The following supporting information can be downloaded at: https://www.mdpi.com/article/10.3390/nu15020358/s1, Figure S1. Kaplan–Meier curves for all-cause mortality according to dietary zinc cutoff points (a) In all, (b) men, and (c) women; Figure S2. Kaplan–Meier curves for cancer mortality according to dietary zinc cutoff points (a) In all, (b) men, and (c) women; Figure S3. Kaplan–Meier curves for cardiovascular mortality according to dietary zinc cutoff points. (a) In all, (b) men, and (c) women. Table S1. Baseline characteristics of study population according to zinc intake (mg/day), Table S2. Baseline characteristics of study population according to zinc intake in men, Table S3. Baseline characteristics of study population according to zinc intake in women, Table S4. Proportions of all-cause, cancer, and cardiovascular disease mortality according to the dietary zinc cutoff points.
