# Supplementary material for: Dietary Zinc Intake and All-Cause and Cardiovascular Mortality in Korean Middle-Aged and Older Adults
Source: Nutrients. 2023 Jan 11;15(2):358. doi: 10.3390/nu15020358 (PMC9862936; doi:10.3390/nu15020358)
Supplement: Supplementary file 1 [file nutrients-15-00358-s001.zip › Supplementary materials.pdf]

Supplementary materials

Dietary zinc intake and all-cause and cardiovascular mortality in Korean middle-aged and older adults

Short title: Dietary zinc intake and mortality

Yu-Jin Kwon,MD,Ph.D[1†]; Hye Sun Lee,Ph.D[12†]; Goeun Park, Ph.D[3]; Juyeon Yang, M.S[2]; Hyung-Mi Kim,Ph.D[14]; Ji-Won Lee MD,Ph.D[5]

[1]Department of Family Medicine, Yongin Severance Hospital, Yonsei University College of Medicine, Gyeonggi, Republic of Korea

[2]Biostatistics Collaboration Unit, Department of Research Affairs, Yonsei University College of Medicine, Seoul, Republic of Korea

[3]Biomedical statistics center, Research institute for future medicine, Samsung medical center

[4]Department of Food and Nutrition, Dongduk Women's University, Seoul 02748, Korea

[5]Department of Family Medicine, Severance Hospital, Yonsei University College of Medicine, Seoul, Republic of Korea

[†]These authors have contributed equally to this work and share first authorship

Corresponding author

Ji-Won Lee, M.D., Ph.D.

Department of Family Medicine, Yonsei University College of Medicine, Severance Hospital, Yonsei-ro 50-1, Seodaemun-gu, Seoul, Republic of Korea, 03722

Tel: +82 2 2019 3480,

Fax: +82 3462 8209,

E-mail: indi5645@yuhs.ac

Table S1. Baseline characteristics of study population according to zinc intake (mg/day) –

**Variables**	**≤5.60**	**5.60–7.98**	**>7.98**	***p*-value**
N	32,992	55,029	55,029	
Sex (men)	8947 (27.1)	19,528 (35.5)	22,469 (40.8)	<.001
Age (years)	55.6±9.3	53.9±8.6	52.7±8.3	<.001
Body mass index (kg/m^2)	23.8±3.0	24.0±2.9	24.1±2.9	<.001
WC (cm)	80.7±8.9	81.3±8.7	81.7±8.8	<.001
SBP (mmHg)	122.9±16.1	122.8±15.4	122.3±15.0	<.001
DBP (mmHg)	76.2±10.2	76.2±10.1	76.3±9.9	.171
Glucose (mg/dl)	95.5±21.4	95.6±21.2	95.7±21.5	.721
HbA1c (%)	5.7±0.8	5.7±0.7	5.7±0.7	.647
TC (mg/dL)	197.7±36.3	196.8±35.6	197.8±35.4	<.001
HDL-C (mg/dL)	52.2±13.2	52.5±13.0	53.0±13.0	<.001
Triglycerides (mg/dL)	129.2±88.4	128.9±90.4	130.1±92.8	.081
Smoking status, n (%)				<.001
Never smoker	25,499 (77.3)	39,813 (72.4)	37,475 (68.1)	
Former smoker	3856(11.7)	8506(15.5)	9523(17.3)	
Current smoker	3637(11.0)	6710(12.2)	8031(14.6)	
Alcohol intake, n (%)				<.001
Never drinker	18,824 (57.1)	28,400 (51.6)	25,148 (45.7)	
Former drinker	1476 (4.5)	2186 (4.0)	2123 (3.9)	
Current drinker	12,692 (38.5)	24,443 (44.4)	27,758 (50.4)	
Regular exercise (Yes)	13,828 (41.9)	27,293 (49.6)	30,608 (55.6)	<.001
Hypertension, n (%)	6051(18.3)	9490 (17.3)	8866 (16.1)	<.001
Diabetes, n (%)	2368 (7.2)	4069 (7.4)	3927(7.1)	0.221
Dyslipidemia, n (%)	18,824 (57.1)	30,904 (56.2)	31,491(57.2)	.001
CKD, n (%)	1394 (4.2)	1425 (2.6)	1073 (2.0)	<.001
Residential area, n (%)				<.001
Urban	25,976 (78.7)	47,960 (87.2)	50,420 (91.6)	
Rural	7016 (21.3)	7069 (12.9)	4609 (8.4)	
Total energy (kcal/day)	1172.9±245.0	1617.9±231.8	2191.0±512.6	<.001
Carbohydrate (g/day)	222.5±54.4	297.8±52.2	373.8±87.4	<.001
Fat (g/day)	14.0±6.6	22.4±8.3	40.3±19.3	<.001
Protein (g/day)	34.7±7.5	51.1±8.3	80.1±25.4	<.001
Calcium (mg/day)	243.2±118.1	375.2±146.5	617.1±293.7	<.001
Phosphorus (mg/day)	542.2±123.0	784.6±135.6	1182.7±350.0	<.001
Iron (mg/day)	5.7±1.8	8.5±2.2	13.7±5.4	<.001

CKD, chronic kidney disease; WC, waist circumference; SBP, systolic blood pressure; DBP, diastolic blood pressure; HbA1C, glycated hemoglobin; HDL-C, high-density lipoprotein cholesterol; TC, total cholesterol

Table S2. Baseline characteristics of study population according to zinc intake in men

Variables	≤5.60	5.60–7.23	>7.23	*p*-value
N	8943	13,500	28,501	
Age (years)	57.0±9.6	55.3±9.0	53.8±8.8	<.001
Body mass index (kg/m^2)	24.0±2.8	24.2±2.7	24.6±2.8	<.001
WC (cm)	84.9±7.8	85.4±7.5	86.2±7.6	<.001
SBP (mmHg)	125.7±15.6	125.6±14.8	125.5±14.4	.581
DBP (mmHg)	78.6±10.2	78.5±9.9	78.7±9.8	.018
Glucose (mg/dl)	99.5±24.9	99.4±23.2	99.8±24.6	.265
HbA1c (%)	5.77±0.81	5.78±0.83	5.77±0.84	.838
TC (mg/dL)	191.4±35.7	192.2±34.9	194.0±34.7	<.001
HDL-C (mg/dL)	47.9±12.2	48.4±12.0	48.8±11.9	<.001
Triglycerides(mg/dL)	150.9±107.5	149.4±105.6	154.5±111.2	<.001
Smoking status, n (%)				<.001
Never smoker	2538 (28.4)	3848 (28.5)	7670 (26.9)	
Former smoker	3476 (38.9)	5551 (41.1)	11,644 (40.9)	
Current smoker	2929 (32.8)	4101 (30.4)	9187 (32.2)	
Alcohol intake, n (%)				<.001
Never drinker	2226 (24.9)	2954 (21.9)	5254 (18.4)	
Former drinker	894 (10.0)	1102 (8.2)	1903 (6.7)	
Current drinker	5823 (65.1)	9444 (70.0)	21,344 (74.9)	
Regular exercise (Yes)	3935 (44.0)	6982 (51.7)	16514 (57.9)	<.001
Hypertension, n (%)	2042 (22.8)	2933 (21.7)	6000 (21.1)	.001
Diabetes, n (%)	836 (9.4)	1360(10.1)	2872(10.1)	.113
Dyslipidemia, n (%)	5131 (57.4)	7725(57.2)	17039(59.8)	<.001
CKD, n (%)	364 (4.1)	403(3.0)	728(2.6)	<.001
Residential area, n (%)				<.001
Urban	6871 (76.8)	11,412 (84.5)	25,478 (89.4)	
Rural	2072 (23.2)	2088 (15.5)	3023 (10.6)	
Total energy (kcal/day)	1242.9±236.5	1592.7±200.5	2136.5±489.1	<.001
Carbohydrate (g/day)	235.7±52.6	294.1±46.0	366.5±82.5	<.001
Fat (g/day)	14.9±6.7	21.7±7.7	38.5±18.1	<.001
Protein (g/day)	35.8±7.1	48.9±6.8	76.0±23.8	<.001
Calcium (mg/day)	216.2±103.5	316.0±120.0	527.3±244.4	<.001
Phosphorus (mg/day)	545.9±114.6	735.1±112.1	1101.2±317.9	<.001
Iron (mg/day)	5.5±1.7	7.8±1.8	12.4±4.7	<.001

P-values are derived from the independent *t*-test for continuous variables, and the chi-square test for categorical variables; significance was set at $p < .05$.

WC, waist circumference; SBP, systolic blood pressure; BP, diastolic blood pressure, TC, Total cholesterol; HDL-C, high-density lipoprotein cholesterol; HbA1c, glycated hemoglobin; CKD, chronic kidney disease

Table S3. Baseline characteristics of study population according to zinc intake in women

Variables	≤5.48	5.48-7.99	>7.99	*p*-value
N	22,098	37,503	32,505	
Age (years)	55.2±9.2	53.4±8.3	52.1±7.9	<.001
Body mass index (kg/m^2)	23.7±3.1	23.8±3.0	23.7±3.0	.004
WC (cm)	79.1±8.7	79.0±8.4	78.6±8.2	<.001
SBP (mmHg)	121.8±16.1	121.3±15.5	120.2±15.1	<.001
DBP (mmHg)	75.2±10.0	74.9±9.9	74.6±9.7	<.001
Glucose (mg/dl)	94.0±19.5	93.6±19.6	92.8±18.4	.001
HbA1c (%)	5.70±0.73	5.69±0.70	5.67±0.66	<.001
TC (mg/dL)	200.1±36.2	199.2±35.8	200.2±35.7	<.001
HDL-C (mg/dL)	53.8±13.2	54.6±13.0	55.9±13.1	<.001
Triglycerides(mg/dL)	121.2±79.1	117.1±77.1	113.0±72.5	<.001
Smoking status, n (%)				<.001
Never smoker	21069(95.3)	36288(96.8)	31374(96.5)	
Former smoker	357(1.6)	441(1.2)	416(1.3)	
Current smoker	672(3.0)	774(2.1)	715(2.2)	
Alcohol intake, n (%)				<.001
Never drinker	15217(68.9)	25653(68.4)	21068(64.8)	
Former drinker	545(2.5)	702(1.9)	639(2.0)	
Current drinker	6336(28.7)	11148(29.7)	10798(33.2)	
Regular exercise (Yes)	9068 (41.0)	17834 (47.6)	17396 (53.5)	<.001
Hypertension, n (%)	3674(16.6)	5562(14.8)	4196(12.9)	<.001
Diabetes, n (%)	1406(6.4)	2255(6.0)	1635(5.0)	.001
Dyslipidemia, n (%)	12548(56.8)	20792(55.4)	17984(55.3)	<.001
CKD, n (%)	968(4.4)	908(2.4)	521(1.6)	<.001
Residential area, n (%)				<.001
Urban	17478(79.1)	32932(87.8)	30185(92.9)	
Rural	4620(20.9)	4571(12.2)	2320(7.1)	
Total energy (kcal/day)	1127.1±238.3	1586.8±235.4	2164.9±520.1	<.001
Carbohydrate (g/day)	214.0±53.4	293.2±53.6	370.7±88.9	<.001
Fat (g/day)	13.4±6.4	21.6±8.2	39.4±19.6	<.001
Protein (g/day)	33.7±7.4	50.4±8.5	79.5±26.0	<.001
Calcium (mg/day)	248.2±119.6	390.5±151.5	654.5±312.8	<.001
Phosphorus (mg/day)	530.9±122.9	783.2±140.5	1195.4±366.5	<.001
Iron (mg/day)	5.9±1.8	8.6±2.3	14.0±5.7	<.001

P-values are derived from the independent *t*-test for continuous variables, and the chi-square test for categorical variables; significance was set at $p < .05$.

WC, waist circumference; SBP, systolic blood pressure; BP, diastolic blood pressure, TC, Total cholesterol; HDL-C, high-density lipoprotein cholesterol; HbA1c, glycated hemoglobin; CKD, chronic kidney disease

Table S4. **Proportions of all-cause, cancer, and cardiovascular disease mortality according to the dietary zinc cutoff points**

	Total		Male		Female	
	Proportion (SD)	p-value	Proportion (SD)	p-value	Proportion (SD)	p-value
All-cause mortality		<.0001		<.0001		<.0001
≤cut1	5.459 (0.125)		10.656 (0.326)		3.638 (0.126)	
cut1–cut2	3.629 (0.08)		6.533 (0.213)		2.197 (0.076)	
>cut2	2.977 (0.072)		5.154 (0.131)		1.551 (0.069)	
Cancer mortality		<.0001		<.0001		<.0001
≤cut1	2.07 (0.078)		3.983 (0.191)		1.237 (0.059)	
cut1–cut2	1.567 (0.043)		2.586 (0.094)		0.949 (0.046)	
>cut2	1.355 (0.073)		2.1 (0.131)		0.76 (0.078)	
CVD mortality		<.0001		<.0001		<.0001
≤cut1	1.209 (0.072)		1.908 (0.159)		0.947 (0.074)	
cut1–cut2	0.74 (0.038)		1.213 (0.089)		0.452 (0.033)	
>cut2	0.481 (0.026)		0.821 (0.053)		0.225 (0.026)	

SD, standard deviation; CVD, cardiovascular disease; cut1; cut2
