# Supplementary figures and images for: Dietary Zinc Intake and All-Cause and Cardiovascular Mortality in Korean Middle-Aged and Older Adults
Source: Nutrients. 2023 Jan 11;15(2):358. doi: 10.3390/nu15020358 (PMC9862936; doi:10.3390/nu15020358)

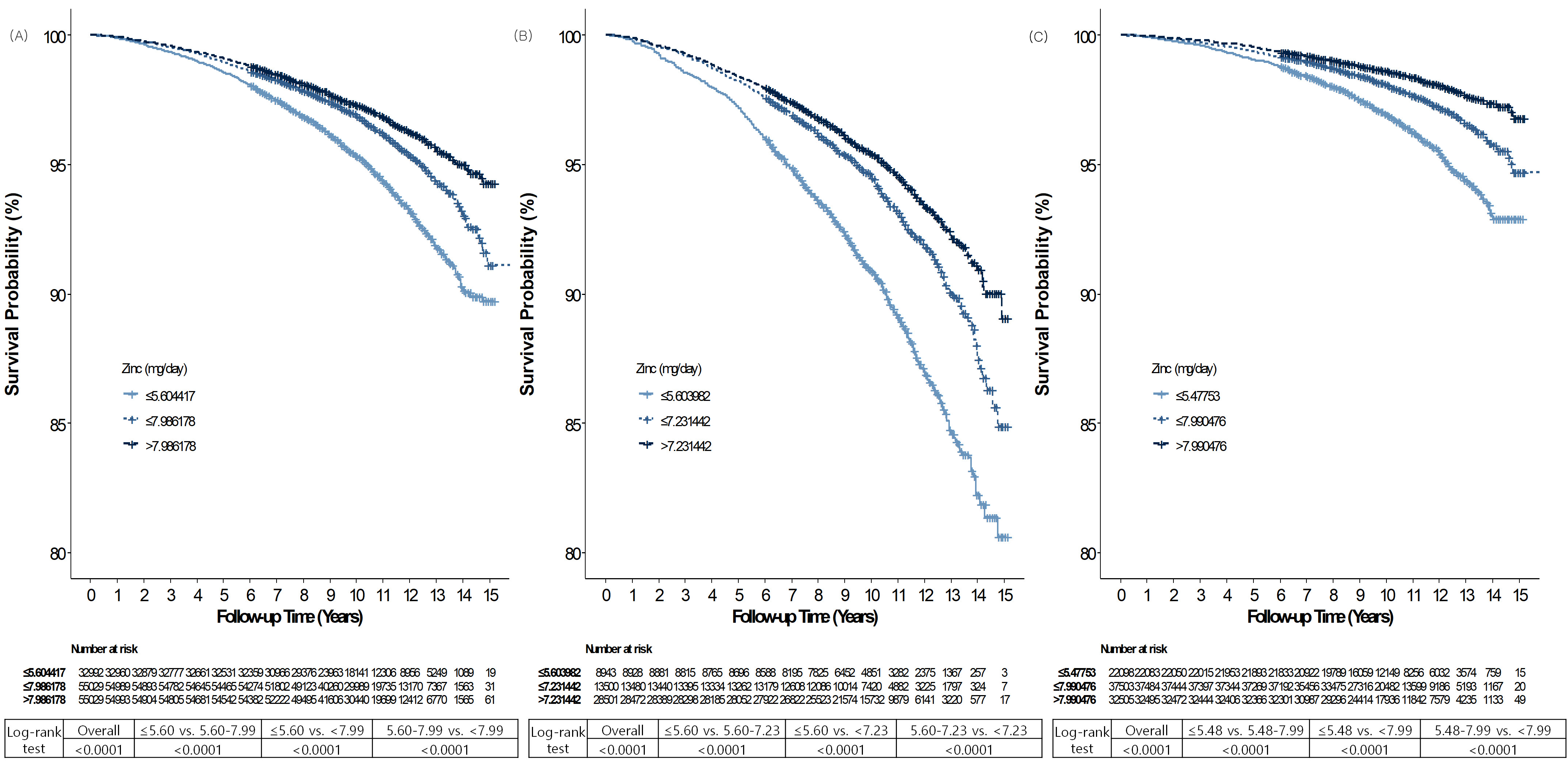

Supplement: Supplementary file 1 [file nutrients-15-00358-s001.zip › FigureS1.tif]

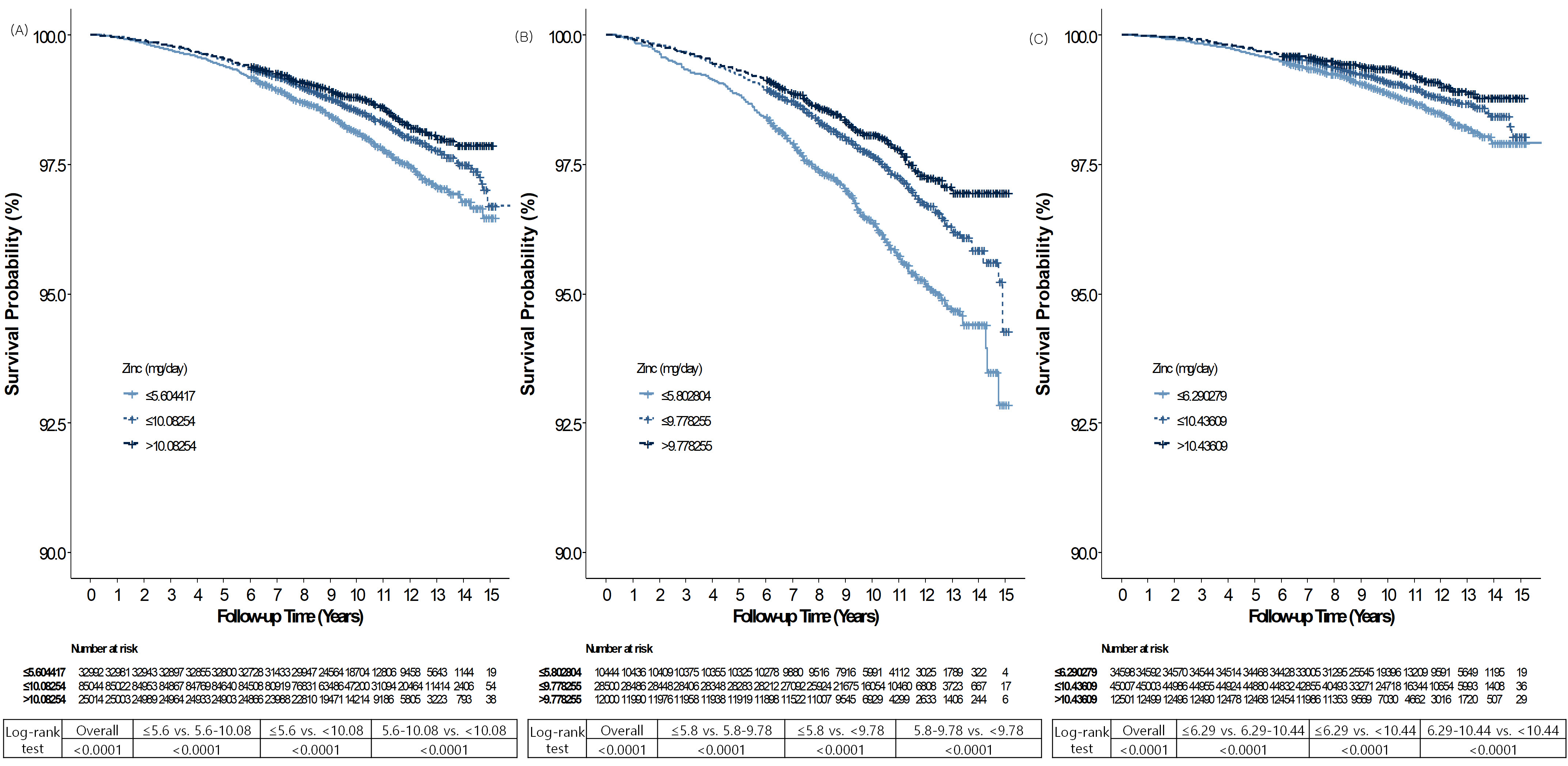

Supplement: Supplementary file 1 [file nutrients-15-00358-s001.zip › FigureS2_new.tif]

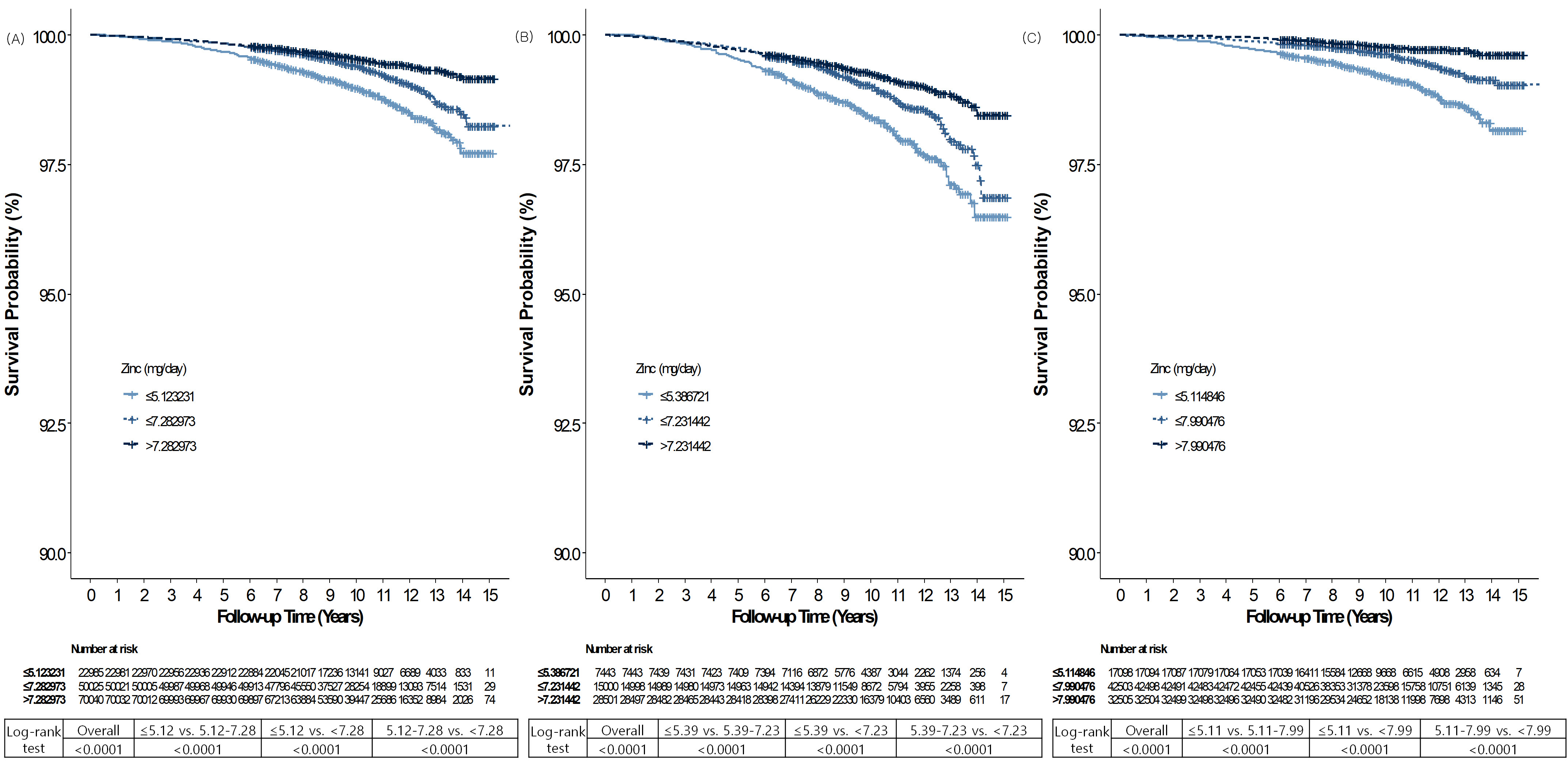

Supplement: Supplementary file 1 [file nutrients-15-00358-s001.zip › FigureS3_NEW.tif]
